# Effect of adjuvant endocrine therapy on recurrence and contralateral breast cancer in HR-positive DCIS after mastectomy

**DOI:** 10.1016/j.breast.2025.104615

**Published:** 2025-10-16

**Authors:** Tae-in Yoon, Ah Yoon Kim, Su Min Lee, Jisun Kim, Il Yong Chung, Beom Seok Ko, Hee Jeong Kim, Jong Won Lee, Byung Ho Son, Seok Jin Nam, Seok Won Kim, Jeong Eon Lee, Jonghan Yu, Woong Ki Park, On Vox Yi, Jai Min Ryu, Sae Byul Lee

**Affiliations:** a Division of Breast Surgery, Department of Surgery, Dongnam Institute of Radiological and Medical Science, Busan, Republic of Korea; b Division of Breast Surgery, Department of Surgery, University of Ulsan College of Medicine, Asan Medical Center, Seoul, Republic of Korea; c Department of Surgery, Gangnam Severance Hospital, Yonsei University College of Medicine, Seoul, Republic of Korea; d Division of Breast Surgery, Department of Surgery, Samsung Medical Center, Sungkyunkwan University School of Medicine, Seoul, Republic of Korea

**Keywords:** Ductal carcinoma in situ, Endocrine therapy, Mastectomy, Tamoxifen, Contralateral breast cancer

## Abstract

**Background:**

The clinical benefits of adjuvant endocrine therapy for patients with hormone receptor (HR)-positive ductal carcinoma in situ (DCIS) undergoing mastectomy remain controversial. While endocrine therapy is known to reduce recurrence after breast-conserving surgery, its role post-mastectomy is unclear. We aimed to assess the impact of adjuvant endocrine therapy on recurrence and contralateral breast cancer (CBC) in patients with HR-positive DCIS treated with mastectomy.

**Methods:**

In this retrospective multicenter cohort study, we included patients with HR-positive, pure DCIS who underwent mastectomy between 2003 and 2018 across three cancer centers in South Korea. Patients were stratified based on receipt of adjuvant endocrine therapy (ETx). Logistic regression and Cox proportional hazards models were used to evaluate associations between ETx and recurrence or CBC. Annual hazard rates were estimated using kernel-smoothed functions.

**Results:**

Of 1,186 eligible patients, 599 (50.5 %) received endocrine therapy. Median follow-up was 86.3 months. The recurrence rate was significantly lower in the ETx group compared to no-ETx (7.0 % vs. 11.7 %; OR, 0.57; p = 0.005). Locoregional recurrence was also lower (2.5 % vs. 4.8 %; OR, 0.51; p = 0.04). CBC occurred in 5.3 % overall, with a non-significant reduction in the ETx group (4.2 % vs. 6.5 %; OR, 0.63; p = 0.08). In multivariable Cox models, ETx was associated with reduced recurrence (HR, 0.53; p = 0.01) and CBC (HR, 0.53; p = 0.04). Risk reduction persisted beyond 10 years.

**Conclusion:**

Adjuvant endocrine therapy was associated with significant reductions in recurrence and CBC risk after mastectomy for HR-positive DCIS, supporting selective use based on individual risk assessment.

## Highlights

• Endocrine therapy reduced recurrence after mastectomy for hormone-positive DCIS.
• Endocrine therapy lowered contralateral breast cancer risk by 47 % in long follow-up.
• Long-lasting benefit of endocrine therapy was seen beyond 10 years after surgery.

## 1 Introduction

Ductal carcinoma in situ (DCIS) is a non-invasive form of breast cancer that is often managed with total mastectomy in cases involving large tumor size, multicentric lesions, the presence of BRCA mutations, or inability to obtain clear margins [1,2]. Total mastectomy offers excellent local control and disease-specific survival for patients with DCIS. Multiple large studies have reported local recurrence rates after TM ranging from 1 % to 4 % over long-term follow-up [3,4]. Despite these favorable outcomes, DCIS survivors remain at risk for subsequent breast events, including contralateral breast cancer (CBC), especially as survival improves and follow-up duration lengthens [[5], [6], [7]].

In patients with hormone receptor-positive DCIS, adjuvant endocrine therapy reduces the risk of both ipsilateral recurrence and new primary contralateral breast cancer after breast-conserving surgery. Large randomized trials such as the NSABP B-24 and UK/ANZ DCIS have demonstrated that tamoxifen reduces the incidence of CBC by approximately 50 % in this population. [6,8,9]. As a result, endocrine therapy is widely recommended for patients with HR-positive DCIS following lumpectomy to reduce the risk of recurrence and contralateral breast cancer [10,11]. However, the role of adjuvant endocrine therapy following mastectomy for DCIS remains controversial, as the absolute risk reduction for ipsilateral recurrence is negligible, and the long-term benefit for contralateral breast cancer is less clearly established. Consequently, substantial variability exists in the clinical application of endocrine therapy after mastectomy [12]. Recent retrospective studies have reported that adjuvant endocrine therapy may not decrease the risk of CBC in patients with unilateral DCIS treated with mastectomy [13,14].

Tamoxifen use is associated with a range of adverse effects, including thromboembolic events, endometrial cancer, and menopausal symptoms [9,15] In recent years, de-escalation strategies for endocrine therapy in early breast cancer and DCIS have gained attention, with an emphasis on personalized treatment approaches based on patient age, risk factors, and risk period. Ongoing research continues to assess the overall benefit-risk balance of tamoxifen in patients with DCIS, and the strength and applicability of endocrine therapy guidelines remain subjects of ongoing clinical debate [11].

To address these uncertainties, we evaluated the effect of adjuvant endocrine therapy on recurrence and contralateral breast cancer risk in patients with HR-positive DCIS who underwent mastectomy, with the aim of providing tailored endocrine therapy recommendations for this patient group.

## 2 Methods

### 2.1 Study design and population

This retrospective cohort study used prospectively collected registry data from three cancer centers in South Korea. Patients diagnosed with ductal carcinoma in situ (DCIS) who underwent total mastectomy between January 2003 and December 2018 were retrospectively screened for eligibility. The median follow-up duration was 86.3 months (interquartile range [IQR], 59.8–120.3 months). Only patients with pathologically confirmed pure DCIS (i.e., without invasive or microinvasive components) were included. Patients were excluded if they had invasive carcinoma, bilateral disease at diagnosis, estrogen receptor (ER) negative or unknown hormone receptor (HR) status, or missing follow-up data. Eligible patients were subsequently stratified based on receipt of adjuvant endocrine therapy. Six male patients were included, but sex-based analyses were not feasible due to their small number. The findings primarily reflect outcomes in female patients.

The study was approved by the institutional review boards of the three participating centers: Asan Medical Center (IRB No.2025-0101), Samsung Medical Center (IRB No.2024-12-158), and Dongnam Institute of Radiological and Medical Science (IRB No. 2506-003-002). The study adhered to the Strengthening the Reporting of Observational Studies in Epidemiology (STROBE) guidelines. The requirement for informed consent was waived because of the retrospective design and the use of anonymized data.

### 2.2 Data collection

Data were extracted from institutional clinical registries and included demographic and clinicopathological characteristics, such as age at diagnosis, body mass index (BMI), family history of breast cancer, BRCA mutation status, type of breast and axillary surgery, tumor size, nuclear grade, and expression level of PR, HER2, and Ki-67. Family history of breast cancer was defined as having at least one first- or second-degree relative diagnosed with breast cancer. Tumor size was defined as the maximum pathological extent of DCIS measured microscopically by pathologists on the final surgical specimens. Patients with any invasive carcinoma or lymph node positivity on final pathology were excluded, ensuring that only pure DCIS cases were included. Hormone receptor (HR)–positive disease was defined as positivity for ER on immunohistochemistry. The treatment data included the administration of adjuvant endocrine therapy (primarily tamoxifen). The decision to initiate endocrine therapy was made by the treating physician in consultation with the patient, considering clinical judgment, patient preferences, and the risks and benefits of therapy. Although the precise duration of endocrine therapy was not available for all patients, the majority were prescribed tamoxifen at a daily dose of 20 mg, with a planned treatment duration of approximately 5 years according to standard clinical practice. Follow-up information included recurrence status, contralateral breast cancer (CBC) diagnosis, date of recurrence, death, and last follow-up.

Overall recurrences were defined as composites of locoregional recurrence, distant metastasis, or CBC. Contralateral breast cancer was defined as a new primary malignancy in the opposite breast diagnosed at least six months after the initial diagnosis of unilateral DCIS, to minimize misclassification with synchronous CBC. Recurrence and CBC-free survival were measured from the date of mastectomy to the date of recurrence and CBC events, respectively.

### 2.3 Statistical analysis

Baseline characteristics were compared using the chi-square test or Fisher's exact test for categorical variables and the Mann–Whitney *U* test for continuous variables. Univariate and multivariate logistic regression analyses were conducted to evaluate associations between endocrine therapy and recurrence outcomes, including locoregional, distant, and contralateral breast cancer (CBC).

Cox proportional hazards models were constructed to identify independent predictors of CBC and recurrence. The covariates included in the multivariate models were age, BMI, family history of breast cancer, tumor size, nuclear grade, HER2 status, and endocrine therapy. Hazard ratios (HRs) and 95 % confidence intervals (CIs) were calculated. The annual hazard rates were estimated according to endocrine therapy status using kernel-smoothed hazard functions. A p-value of <0.05 was considered statistically significant. All analyses were performed using R software (version 4.5.0; R Foundation for Statistical Computing, Vienna, Austria).

## 3 Results

### 3.1 Patient characteristics

Of the 1,572 patients initially identified, 386 were excluded based on predefined eligibility criteria, resulting in a final cohort of 1,186 patients with hormone receptor (HR)-positive DCIS. Patients were classified into two groups based on the receipt of adjuvant endocrine therapy: ETx (n = 599) and no-ETx (n = 587). Clinicopathological characteristics were compared between the two groups (Table 1).

**Table 1:** Baseline clinicopathological characteristics of patients.

Variable		Total (N = 1186)	No ETx (N = 587)	ETx (N = 599)	p-value
Age at diagnosis (years)	Median (IQR)	46(41–51)	46(40–51)	46(41–51)	.97
BMI	<18.5	86 (7.3 %)	41 (7.1 %)	45 (7.5 %)	.90
	18.5–24.9	867 (73.5 %)	430 (74.1 %)	437 (73.0 %)	
	≥25	226 (19.2 %)	109 (18.8 %)	117 (19.5 %)	
	Unknown	7	7	0	
Family History	No	1027 (87.3 %)	505 (87.4 %)	522 (87.3 %)	1.00
	Yes	149 (12.7 %)	73 (12.6 %)	76 (12.7 %)	
	Unknown	10	9	1	
BRCA Mutation	Wild	153 (91.1 %)	71 (85.5 %)	82 (96.5 %)	.03
	BRCA1/2 mutation	15 (8.9 %)	12 (14.5 %)	3 (3.5 %)	
	Unknown	1018	504	514	
Axillary surgery	No	50 (4.2 %)	35 (6.0 %)	15 (2.5 %)	<.001
	SLNB	955 (80.5 %)	430 (73.3 %)	525 (87.6 %)	
	ALND	181 (15.3 %)	122 (20.8 %)	59 (9.8 %)	
Tumor size	<5 cm	770 (65.4 %)	449 (77.1 %)	321 (53.9 %)	<.001
	≥5 cm	408 (34.6 %)	133 (22.9 %)	275 (46.1 %)	
	Unknown	8	5	3	
Nuclear grade	1,2	970 (85.6 %)	483 (87.8 %)	487 (83.5 %)	.05
	3	163 (14.4 %)	67 (12.2 %)	96 (16.5 %)	
	Unknown	53	37	16	
PR	Negative	119 (10.0 %)	85 (14.5 %)	34 (5.7 %)	<.001
	Positive	1066 (90.0 %)	501 (85.5 %)	565 (94.3 %)	
	Unknown	1	1	0	
HER2	Negative	742 (81.0 %)	342 (79.7 %)	400 (82.1 %)	.40
	Positive	174 (19.0 %)	87 (20.3 %)	87 (17.9 %)	
	Unknown	270	158	112	
Ki-67	<15 %	650 (76.2 %)	243 (78.9 %)	407 (74.7 %)	.19
	≥15 %	203 (23.8 %)	65 (21.1 %)	138 (25.3 %)	
	Unknown	333	279	54	

ETx, endocrine therapy; PR, progesterone receptor; HER2, human epidermal growth factor receptor 2; SLNB, sentinel lymph node biopsy; ALND, axillary lymph node dissection.

The median age at diagnosis was 46 years (IQR, 41–51) in both groups. The no-ETx group had a higher proportion of PR-negative (14.5 % vs. 5.7 %, p < 0.001) tumors. BRCA mutation testing was conducted in 168 patients (14.1 %), with mutations detected more frequently in the no-ETx group than in the ETx group (14.5 % vs. 3.5 %, p = 0.03).

Tumors ≥5 cm were more common in the ETx group (46.1 % vs. 22.9 %, p < 0.001). Axillary surgery, defined as sentinel lymph node biopsy (SLNB) or axillary lymph node dissection (ALND), was performed more often in the ETx group (97.5 % vs. 94.0 %, p < 0.001).

### 3.2 Recurrence and contralateral breast cancer outcomes

**Fig. 1:**
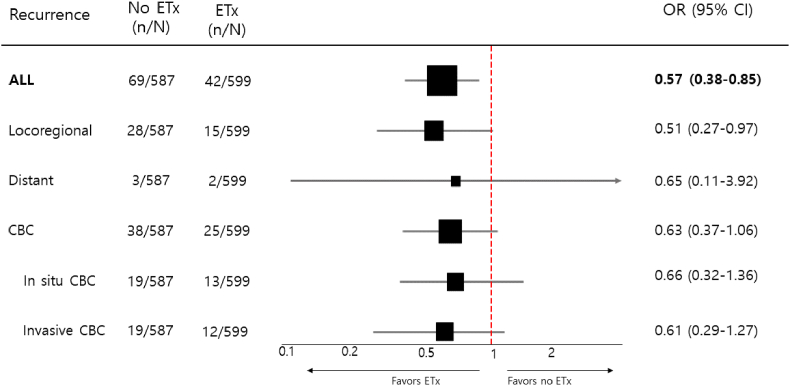
Effect of Adjuvant Endocrine Therapy on Recurrence and Contralateral Breast Cancer in HR-positive DCIS ETx, Endocrine therapy; OR, Odds ratio; CBC, Contralateral breast cancer.

**Fig. 2:**
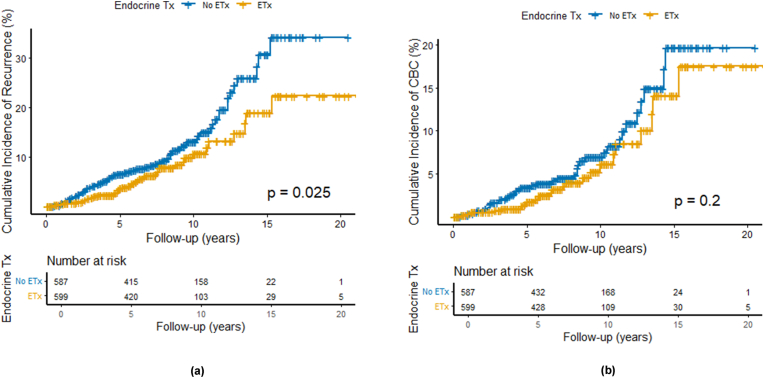
Kaplan-Meier curve for cumulative incidence of Recurrence(A) and Contralateral breast cancer(B) by Endocrine Therapy Group ETx, Endocrine therapy.

A total of 111 recurrence events (9.4 %) were observed in 1,186 patients. The median time to local recurrence was 64.0 months (IQR, 33.5–109.0). The overall recurrence rate was significantly lower in the ETx group than in the no-ETx group (7.0 % vs. 11.7 %; odds ratio [OR], 0.57; 95 % confidence interval [CI], 0.38–0.85; p = 0.005). Locoregional recurrence also occurred less frequently in the ETx group (2.5 % vs. 4.8 %; OR, 0.51; 95 % CI, 0.27–0.97; p = 0.04) (Fig. 1). Kaplan–Meier analysis revealed a significantly lower cumulative incidence of overall recurrence in the ETx group (log-rank p = 0.025; Fig. 2A). The 5- and 10-year cumulative incidences of overall recurrence were 3.6 % and 10.7 % in the ETx group and 6.6 % and 13.0 % in the no-ETx group.

Contralateral breast cancer (CBC) was diagnosed in 64 patients (5.3 %), with a lower incidence observed in the ETx group than in the no-ETx group (4.2 % vs. 6.5 %), although this difference did not reach statistical significance (OR, 0.63; 95 % CI, 0.37–1.06; p = 0.08). The median time to CBC was 70.0 months (IQR, 41.5–121.5). At 5 and 10 years, the cumulative incidences of CBC were 1.7 % and 6.1 % in the ETx group and 3.4 % and 6.9 % in the no-ETx group. In subgroup analyses, both in situ and invasive CBC occurred less frequently in the ETx group (OR for in situ: 0.66; 95 % CI, 0.32–1.36; p = 0.25; OR for invasive: 0.61; 95 % CI, 0.29–1.27; p = 0.18), though these differences were also not statistically significant (Fig. 1). Similarly, the Kaplan–Meier curve for CBC showed a non-significant trend toward reduced CBC incidence in the ETx group (log-rank p = 0.20; Fig. 2B).

In the multivariable Cox proportional hazards model, adjuvant endocrine therapy was independently associated with a reduced risk of CBC (hazard ratio [HR], 0.53; 95 % CI, 0.29–0.98; p = 0.04) and overall recurrence (HR, 0.53; 95 % CI, 0.33–0.84; p = 0.01). Increased age was also associated with a lower risk of recurrence (HR, 0.97; 95 % CI, 0.96–0.98; p < 0.001). No other clinicopathological factors, including BMI, family history, tumor size, nuclear grade, or HER2 status, were significantly associated with either CBC or recurrence (Table 2).

**Table 2:** Multivariate cox proportional hazards analysis for recurrence and CBC in hormone receptor-positive patients.

Variable		Recurrence			CBC		
		HR	95 % CI	p-value	HR	95 % CI	p-value
Age (continuous)		0.97	0.96 to 0.98	< .001	1	0.97 to 1.04	.81
BMI	18.5–24.9 vs < 18.5 (ref)	1.25	0.55 to 2.86	.59	0.71	0.25 to 2.04	.52
	≥25 vs < 18.5 (ref)	1.4	0.52 to 3.75	.50	1.35	0.43 to 4.26	.61
Family history	Yes vs No (ref)	0.91	0.46 to 1.82	.79	1.45	0.64 to 3.23	.37
Tumor size	≥5 cm vs < 5 cm (ref)	1.07	0.66 to 1.73	.79	1.12	0.60 to 2.13	.72
Nuclear grade	3 vs 1,2 (ref)	1.16	0.63 to 2.14	.63	0.61	0.22 to 1.72	.36
HER2 status	Positive vs Negative (ref)	0.77	0.43 to 1.39	.39	0.58	0.24 to 1.41	.23
Endocrine therapy	Yes vs No (ref)	0.53	0.33 to 0.84	.01	0.53	0.29 to 0.98	.04

CBC, Contralateral breast cancer; HR, hazard ratio; BMI, body mass index.

**Fig. 3:**
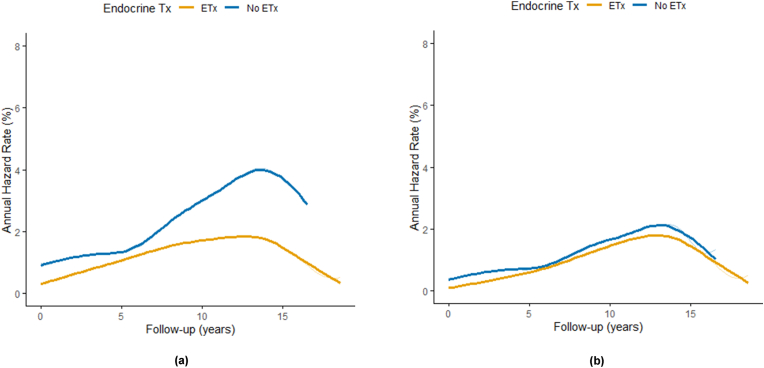
Annual hazard rate curves for (A) Recurrence and (B) Contralateral breast cancer in patients with hormone receptor-positive ductal carcinoma in situ, stratified by endocrine therapy status. ETx, Endocrine therapy.

The annual hazard rates for both recurrence and CBC were consistently lower in the ETx group than those in the no-ETx group, with the peak hazard observed between 10 and 15 years after surgery (Fig. 3). No cancer-specific deaths were observed during the follow-up period.

## 4 Discussion

This multicenter retrospective cohort study provides new evidence that adjuvant endocrine therapy may reduce both recurrence and contralateral breast cancer (CBC) in patients with hormone receptor (HR)-positive ductal carcinoma (DCIS) following mastectomy, and that its use is associated with a significantly lower overall risk of these events. In multivariate analyses, the ETx group showed a 47 % reduction in recurrence risk and CBC risk. Annual hazard plots revealed that the protective effect of endocrine therapy persisted throughout the long-term follow-up period, with the most pronounced risk difference observed between 10 and 15 years postoperatively.

Several prior studies have demonstrated the efficacy of tamoxifen in reducing both ipsilateral recurrence and contralateral breast cancer in patients with HR-positive DCIS, particularly those treated with breast-conserving surgery. Randomized trials, such as the NSABP B-24 and the UK/ANZ DCIS trials, have reported a nearly 50 % reduction in CBC risk with tamoxifen use [6,8,9]. However, the clinical benefit of endocrine therapy following mastectomy remains a matter of ongoing debate. In a large multicenter cohort study of Chinese patients, Niu et al. [13] reported no significant improvement in disease-free survival with adjuvant endocrine therapy after mastectomy, despite including high-risk subgroups. Adverse events occurred in more than one-third of patients who received endocrine therapy, raising concerns regarding potential overtreatment in this population. Additionally, a retrospective study of 176 patients who underwent unilateral mastectomy found no significant difference in the incidence of CBC between those who received endocrine therapy and those who did not, even after 12 years of follow-up [14].

The discrepancy between our findings and those of earlier retrospective studies may be attributable to differences in sample size, statistical power, and event rates. Previous studies were based on relatively small cohorts and observed few CBC or recurrence events, which may have limited their ability to detect statistically significant differences. In contrast, our study comprised a substantially larger cohort with more observed recurrence and CBC events, enabling more robust, adequately powered, and adjusted analyses.

Two major randomized trials, NSABP B-35 and IBIS-II DCIS, compared tamoxifen and aromatase inhibitors (AIs) in postmenopausal women with hormone receptor (HR)-positive DCIS. In the NSABP B-35 trial, anastrozole demonstrated superior efficacy in reducing breast cancer events, particularly among women under the age of 60 years, and was associated with fewer thromboembolic complications [15]. In contrast, the IBIS-II DCIS trial reported no statistically significant difference in recurrence rates between anastrozole and tamoxifen, although non-inferiority of anastrozole was established [16]. In our study, the vast majority of patients who received endocrine therapy were treated with tamoxifen at a standard dose of 20 mg daily for 5 years, as aromatase inhibitors are not reimbursed for DCIS under the Korean national insurance system. Tamoxifen and aromatase inhibitors exhibit distinct adverse effect profiles: Aromatase inhibitors are more frequently associated with musculoskeletal and cardiovascular toxicity, whereas tamoxifen is linked to higher rates of gynecologic and thromboembolic complications [15,16]. A phase III trial also investigated the efficacy of low-dose tamoxifen (5 mg/day), which showed promise in reducing recurrence while minimizing adverse events in women with intraepithelial neoplasia [17].

Considering the heterogeneity of endocrine agents, variations in dosing strategies, and distinct toxicity profiles, these findings underscore the importance of a personalized approach to adjuvant endocrine therapy in patients with DCIS treated with mastectomy. Treatment decisions should be guided by a comprehensive assessment of the recurrence risk, menopausal status, anticipated side effects, treatment adherence, and individual patient preferences. In this context, gene expression profiling tools, such as the Oncotype DX DCIS Score, have been introduced to aid in risk stratification and individualized treatment decision-making, particularly in patients undergoing breast-conserving surgery [18,19]. Although initially developed to predict local recurrence and guide radiotherapy, emerging evidence suggests that the DCIS score may also inform endocrine therapy decisions. However, its clinical validity and utility in post-mastectomy settings remain unclear. Further studies are needed to determine whether genomic assays can refine endocrine therapy recommendations in this population and identify patients who may benefit from treatment de-escalation without compromising long-term outcomes.

An important aspect of this study was the use of annual hazard rate analysis to evaluate time-dependent recurrence and CBC risk. In our cohort, the ETx group consistently showed lower annual hazards, with the most pronounced difference emerging at 10–15 years postoperatively. Supporting this observation, a population-based SEER analysis of over 800,000 women reported a cumulative 25-year contralateral breast cancer risk of approximately 10 % among DCIS patients, with persistent or rising risk beyond 10 years, especially in those with estrogen receptor-positive disease [20]. This highlights the importance of extended risk assessment and supporting the potential role of endocrine therapy in reducing late CBC events.

## 5 Limitations and strengths

This study has several limitations to consider. First, the retrospective nature of the study introduces potential selection bias and susceptibility to unmeasured confounding variables. Second, BRCA mutation testing was conducted only in a minority of patients, limiting our ability to assess its role as a confounder in CBC risk analyses. Third, detailed data on endocrine therapy adherence, duration, and treatment-related toxicities were not available, precluding the assessment of treatment compliance and tolerability. Finally, despite adjustment through multivariate analysis, the possibility of residual confounding cannot be fully excluded.

Despite these limitations, this study has several strengths. First, it included a large, pathologically confirmed cohort of patients with HR-positive DCIS treated with mastectomy, representing one of the largest analyses of endocrine therapy in this population. Second, the multicenter design, which incorporated data from three specialized cancer centers, allowed for a larger cohort and enhanced the generalizability of the findings. Third, access to structured, prospective cancer registries facilitated detailed data collection on recurrence and CBC, supporting robust time-to-event analyses over extended follow-up. Notably, the use of annual hazard function analysis offers novel insight into the temporal dynamics of recurrence and CBC risk, an area seldom addressed in prior DCIS research.

## 6 Conclusion

In patients with hormone receptor-positive DCIS treated with total mastectomy, adjuvant endocrine therapy was significantly associated with reductions in both overall recurrence and contralateral breast cancer. The protective effect of endocrine therapy persisted over long-term follow-up, with annual hazard analyses demonstrating sustained risk reduction, particularly beyond 10 years after surgery. These findings support the selective use of adjuvant endocrine therapy, even among patients undergoing mastectomy for HR-positive DCIS. However, given the overall favorable prognosis after mastectomy and the potential side effects of endocrine therapy, a tailored approach that considers the type of agent, dosage, treatment duration, and selective use in high-risk patients is warranted. Future studies are warranted to refine risk-adapted endocrine therapy strategies and to identify patients who may benefit from de-escalation without compromising outcomes.

## CRediT authorship contribution statement

**Tae-in Yoon:** Writing – review & editing, Writing – original draft, Methodology, Investigation, Formal analysis, Conceptualization. **Ah Yoon Kim:** Investigation, Formal analysis, Data curation, Conceptualization. **Su Min Lee:** Resources, Formal analysis, Data curation, Conceptualization. **Jisun Kim:** Investigation, Funding acquisition. **Il Yong Chung:** Resources, Project administration, Methodology. **Beom Seok Ko:** Supervision, Resources, Data curation. **Hee Jeong Kim:** Validation, Supervision, Investigation, Conceptualization. **Jong Won Lee:** Validation, Supervision. **Byung Ho Son:** Supervision. **Seok Jin Nam:** Project administration, Formal analysis, Data curation. **Seok Won Kim:** Validation, Supervision, Data curation. **Jeong Eon Lee:** Visualization, Validation, Supervision, Data curation, Conceptualization. **Jonghan Yu:** Writing – review & editing, Supervision, Formal analysis, Data curation. **Woong Ki Park:** Software, Resources, Project administration. **On Vox Yi:** Visualization, Methodology. **Jai Min Ryu:** Validation, Software, Formal analysis, Data curation, Conceptualization. **Sae Byul Lee:** Writing – review & editing, Writing – original draft, Supervision, Funding acquisition, Formal analysis, Data curation, Conceptualization.

## Data availability statement

The data supporting the findings of this study are securely maintained in institutional repositories and are not publicly available due to patient privacy considerations. However, de-identified data may be made available from the corresponding author upon reasonable request and with appropriate institutional approvals.

## Funding/support

No funding

## Declaration of competing interests

The authors declare that they have no known competing financial interests or personal relationships that could have appeared to influence the work reported in this paper.
